# An ‘activator-repressor’ loop controls the anthocyanin biosynthesis in red-skinned pear

**DOI:** 10.1186/s43897-024-00102-6

**Published:** 2024-07-01

**Authors:** Guangyan Yang, Zhaolong Xue, Kui Lin-Wang, Guosong Chen, Yongqi Zhao, Yaojun Chang, Shaozhuo Xu, Manyi Sun, Cheng Xue, Jiaming Li, Andrew C. Allan, Richard V. Espley, Jun Wu

**Affiliations:** 1 College of Horticulture, State Key Laboratory of Crop Genetics and Germplasm Enhancement, Nanjing Agricultural University, Nanjing, 210095 China; 2 Zhongshan Biological Breeding Laboratory, Nanjing, 210014 Jiangsu China; 3 The New Zealand Institute for Plant & Food Research Limited, Auckland, 1025 New Zealand; 4 State Key Laboratory of Crop Biology, College of Horticulture Science and Engineering, Shandong Agricultural University, Tai’an, 271018 China

**Keywords:** Pear, Anthocyanin, Repressor, MYB, Transcriptional regulation

## Abstract

The color of red-skinned pear (*Pyrus* spp.) is primarily attributed to accumulation of anthocyanins, which provide nutritional benefits for human health and are closely associated with the commercial value of fruits. Here, we reported the functional characterization of a R2R3-MYB repressor PyMYB107, which forms an ‘activator-repressor’ loop to control anthocyanin accumulation in the red-skinned pear. *PyMYB107* overexpression inhibited anthocyanin biosynthesis in both pear calli and fruits, while virus-induced gene silencing of *PyMYB107* increased anthocyanin accumulation in pear fruits. Furthermore, ectopic expression of *PyMYB107* decreased anthocyanin accumulation in tomato, strawberry and tobacco. PyMYB107 can competitively bind to PybHLH3 with PyMYB10/MYB114, thereby suppressing the transcriptional activation of key anthocyanin biosynthesis genes, *PyANS* and *PyUFGT*. Site-directed mutagenesis showed that mutations within the R3 domain and EAR motif of PyMYB107 eliminated its repressive activity. Additionally, *PyMYB107* exhibited a comparable expression pattern to *PyMYB10*/*MYB114* and was transcriptionally activated by them. Our finding advanced comprehension of the repression mechanism underlying anthocyanin accumulation, providing valuable molecular insights into improving quality of pear fruits.

**Supplementary Information:**

The online version contains supplementary material available at 10.1186/s43897-024-00102-6.

## Core

The transcription of the repressor PyMYB107 could be activated by the activators PyMYB10/MYB114 in red-skinned pear fruits, and PyMYB107 in turn competitively bound to PybHLH3 with PyMYB10/MYB114, thereby preventing overaccumulation of anthocyanins. However, mutations within the R3 domain and EAR motif of PyMYB107 eliminated its repressive activity.

## Gene *&* accession numbers

Information of genes in this study can be found in the database (http://peargenome.njau.edu.cn/) under the accession numbers: *PyMYB107* (Pbr028725.1); *PyMYB10* (Pbr016663.1); *PyMYB114* (Pbr042924.1); *PybHLH3* (Pbr017379.1); *PyTTG1* (Pbr038760.1).

## Introduction

Anthocyanins are natural water-soluble pigments that endow fruits with vibrant color and possess diverse physiological function, including UV radiation resistance, plant pathogen defense, and reactive oxygen species scavenging in plants (Winkel-Shirley 2001; Albert et al. 2014). Moreover, anthocyanins are crucial indicators of fruit maturity and quality, while also offering a valuable source of antioxidants for human health. Therefore, enhancement or modification of fruit coloration is often a primary objective of breeding programs in diverse fruit (Einbond et al. 2004; Allan et al. 2008; Espley and Jaakola 2023).

Anthocyanin biosynthesis is regulated by numerous transcription factors (TFs). One well-studied family of transcription factors controlling anthocyanin biosynthesis are the MYBs (Allan et al. 2008; Allan and Espley 2018). MYB transcription factors (TFs) exhibit versatility, being expressed in all eukaryotes, and possess a diverse range of function (Ambawat et al. 2013). In plants, most MYB proteins possess either one R (R3-MYB) or two R repeat sequences (R2R3-MYB), where a conserved bHLH interaction motif [D/E]Lx2[R/K]x3Lx6Lx3R is located. This motif enables MYBs to interact with bHLH proteins to form the regulatory network MYB-bHLH-WDR (MBW) complex (Chen et al. 2019; Ma and Constabel 2019). The MBW complex is considered to be the central regulatory network involved in anthocyanin biosynthesis (Gonzalez et al. 2008; Albert et al. 2014; Zhao et al. 2023). Examples include blueberry VcMYBA and VcbHLH3 (Plunkett et al. 2018; Lafferty et al. 2022); kiwifruit AcMYB110 and AcbHLH3 (Peng et al. 2019; Herath et al. 2020); peach PpMYB10 and PpbHLH3 (Tuan et al. 2015; Zhou et al. 2015, 2018); apple MdMYB10 and MdbHLH3/bHLH33 (Espley et al. 2007, 2009), and pear PyMYB10/MYB114 and PybHLH3 (Zhai et al. 2016; Yao et al. 2017; Ni et al. 2023);

In addition to activators, MYB proteins also include R2R3-MYB and R3-MYB repressors that negatively regulate the expression of anthocyanin biosynthesis structural genes (Grotewold et al. 2000; Zimmermann et al. 2004; Chen et al. 2019; Ma and Constabel 2019). The repressive activity of the R2R3-MYB repressor is commonly ascribed to its C-terminal inhibitory motifs known as C1 (LIsrGIDPxT/SHRxI/L) and C2 (pdLNLD/ELxiG/S). The C1 motif is also referred to as the GIDP motif, and the C2 domain is known as the EAR domain, which contains a core sequence of LxLxL or DLNxxP. Additionally, some R2R3-MYB repressors possess the TLLLFR repressive motif (Chen et al. 2019; Ma and Constabel 2019; LaFountain and Yuan 2021). Removal of these repressive motifs could result in a reduction or loss of repressive activities. For examples, PhMYB27, PtrMYB57 and MdMYB16 from petunia, poplar and apple respectively, suppressed the expression of flavonoid biosynthetic genes through the EAR domain, while deletion of the EAR domain weakened their repressive activities (Albert et al. 2011; Wan et al. 2017; Xu et al. 2017). Likewise, poplar PtMYB165, PtMYB182 and PtMYB194 can compete with MYB activators for interacting with bHLH proteins to repress anthocyanin and proanthocyanin (PA) accumulation, while mutations within their C-terminal repressive motifs resulted in a reduction of, or loss of, their repressive activities (Yoshida et al. 2015; Ma et al. 2018). In contrast with the R2R3-MYB repressors, R3-MYB repressors contain only one R3 domain or both partial R2 domains, but lack any repressive motifs (Li et al. 2016; Chen et al. 2019; Ma and Constabel 2019; LaFountain and Yuan 2021), such as AtCPC, AtTRY, AtMYBL2, PtrRML1, and LlMYBL1 (D’Amelia et al. 2020; Zhang et al. 2020; Xu et al. 2023). The tomato *SlMYBATV* encodes an R3-MYB transcription factor, and studies have shown that the expression of anthocyanin structural genes and MYB TFs significantly was increased in *SlMYBATV* mutant lines (Cao et al. 2017).

A total of 185 R2R3-MYB genes were identified in the Chinese white pear genome (Li et al. 2016). Among them, PyMYB10 and PyMYB114 were crucial activators that promote anthocyanin biosynthesis. Recently, several R2R3-MYB repressors were reported involved in the regulation of anthocyanin accumulation. For examples, PbMYB120 was able to reduce *PbUFGT1* expression (Song et al. 2020), while PpMYB140 inhibited anthocyanin accumulation through competition with PpMYB114 for interacting with PpbHLH3 (Ni et al. 2021). In additional, co-transformation of the repressor PyMYB73 with PyMYB10/MYB114-PybHLH3 increased anthocyanin accumulation, while co-overexpression of PyMYB6 with PyMYB10/MYB114-PybHLH3 led to a reduction in anthocyanin accumulation. These two repressors were able to regulate anthocyanin accumulation by competitive combination the regulatory complex of PyMYB10/MYB114-PybHLH3 (Yao et al. 2023). These findings suggest a complex regulatory mechanism behind MYB repressors. Although MYB repressors associated with anthocyanin biosynthesis have been reported, the repressive mechanism requires further investigation.

After mining our previously published transcriptome (Liu et al. 2021), an R2R3-MYB repressor, named PyMYB107, was found to be differentially expressed in the pigmented and non-pigmented red-skinned pear fruits, suggesting a potential involvement of PyMYB107 in anthocyanin biosynthesis. In this study, RT-qPCR analysis showed a significantly higher expression level of *PyMYB107* in the skin of ‘Red Zaosu’ pear fruit compared to ‘Zaosu’. Molecular biology techniques were employed to validate that PyMYB107 competed with MYB activators PyMYB10 and PyMYB114 for interaction with PybHLH3, inhibiting the transcription activation of *PyANS* and *PyUFGT*, thereby repressing anthocyanin biosynthesis. Furthermore, the repressive activity of PyMYB107 was inactivated when the EAR motif and R3 domain were mutated. Additionally, the expression of *PyMYB107* is mediated by the activators PyMYB10 and PyMYB114, establishing an ‘activation—inhibition’ regulatory loop in the control of anthocyanin biosynthesis. Our findings provide insight into a potential mechanism for improving fruit quality by promoting anthocyanin content.

## Results

### Expression of *PyMYB107* is associated with anthocyanin accumulation in pear

**Fig. 1:**
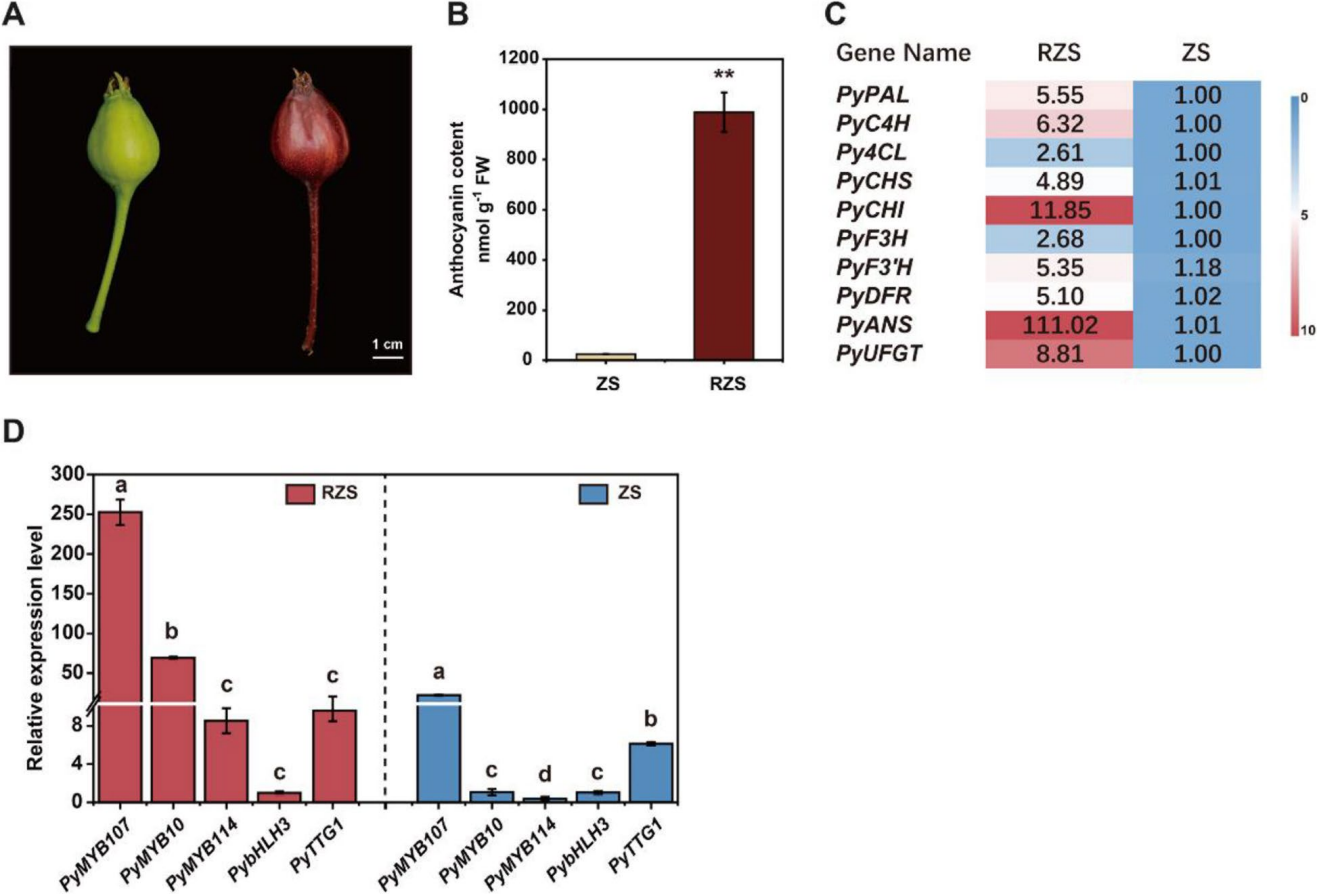
Color phenotype of pear fruits and expression level of the anthocyanin-related genes. **A** Color phenotype of pear fruits of ‘Zaosu’ (ZS) (left) and ‘Red Zaosu’ (RZS) (right). Fruits were collected at 30 days after full bloom (DAFB). **B** Anthocyanin concentration in pear fruit skins. FW, fresh weight. **C** A heatmap showing the expression patten of 10 anthocyanin biosynthetic genes in pear fruits based on RT–qPCR. **D** The transcription level of *PyMYB107*, *PyMYB10*, *PyMYB114*, *PybHLH3* and *PyTTG1* in RZS and ZS. Error bars indicate mean ± SE of three biological replicates. Asterisks indicate the significant difference analyzed by two-tailed Student’s *t*-test (**P < 0.01). Lowercase letters above the bars show the statistical significance analyzed by One-way ANOVA followed by Tukey’s multiple comparisons test (*P* < 0.05)

‘Red Zaosu’ pear is a red bud mutant of ‘Zaosu’ (*P. pyrifolia* × *P. communis)*, characterized by its ability to accumulate anthocyanin in the fruit skin at the young stage (Qian et al. 2014), with higher transcripts of anthocyanin biosynthesis genes (Fig. 1A and B). We have previously demonstrated that PyMYB10 and PyMYB114 play a pivotal role in controlling anthocyanin accumulation of pear fruit skin (Yao et al. 2017; Liu et al. 2021; Yang et al. 2024). In this study, we confirmed a strong correlation between anthocyanin accumulation and the transcript levels of anthocyanin structural genes and MYB activators *PyMYB10* and *PyMYB114* in ‘Red Zaosu’ (Fig. 1C and D; Qian et al. 2014; Sun et al. 2023). By mining our previous transcriptome, we identified a differentially expressed R2R3-MYB repressor, namely PyMYB107, between the pigmented and non-pigmented pear fruits (Liu et al. 2021). We observed a higher expression level of *PyMYB107* in fruit skins of ‘Red Zaosu’ as compared to ‘Zaosu’, and its expression pattern simulates that of *PyMYB10* and *PyMYB114* in ‘Red Zaosu’ (Fig. 1D), suggesting that *PyMYB107* may play a role in the regulation of anthocyanin accumulation in pear.

**Fig. 2:**
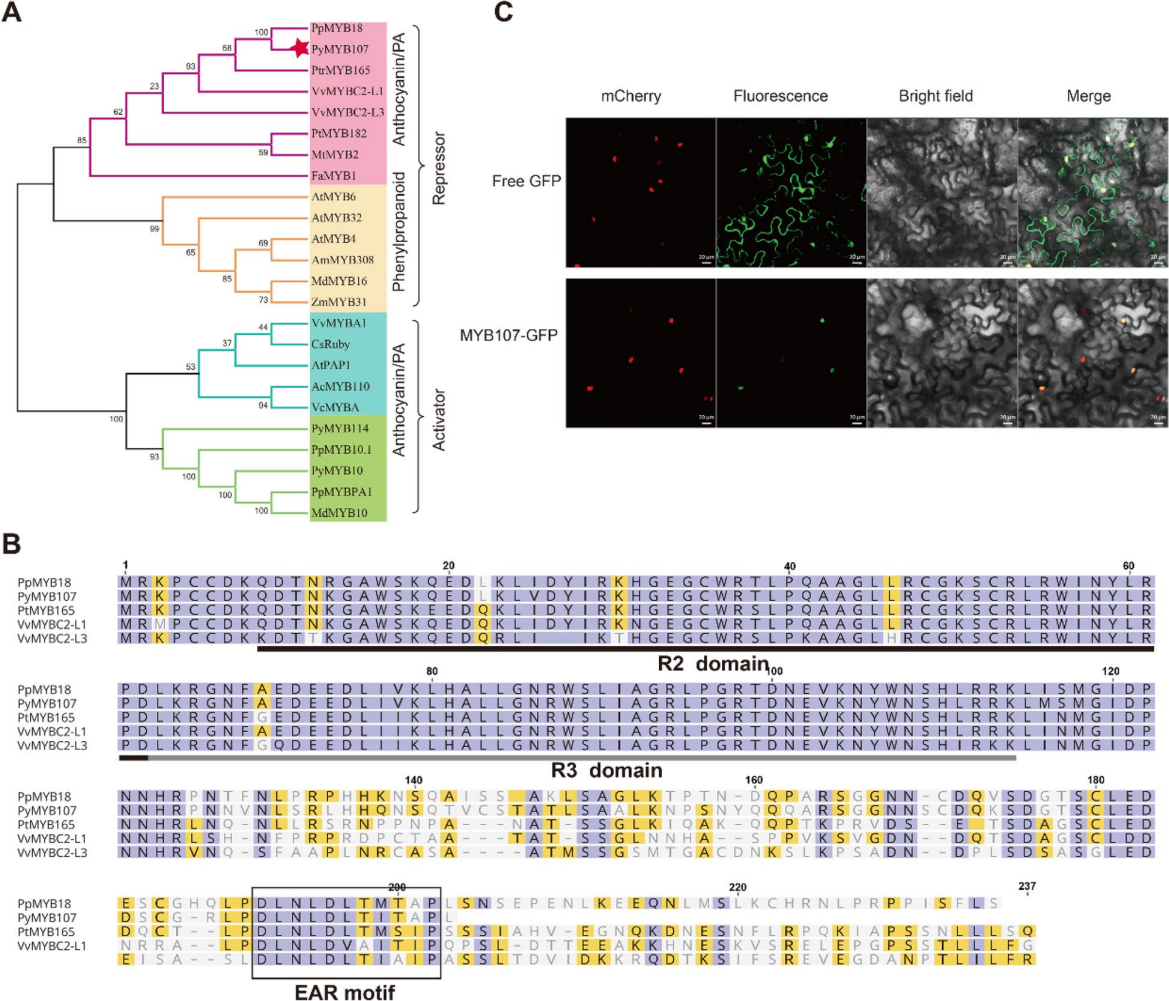
PyMYB107 protein and subcellular localization. **A** Neighbor-joining phylogenetic tree of PyMYB107 and other known anthocyanin-related MYB transcription factors. **B** Alignment of amino acid sequences of PyMYB107, PpMYB18, PtMYB165, VvMYBC2-L1 and VvMYBC2-L3. R2 and R3 domains are underlined. The potential functional EAR motif is boxed. **C** PyMYB107 is a nuclear localized protein, as detected by laser confocal microscopy. Scale bar, 20 μm

The *PyMYB107* gene encodes an R2R3-MYB protein consisting of 203 amino acid residues. PyMYB107 is a member of the S4 subgroup of the R2R3-MYB family (Li et al. 2016), and shares phylogenetic similarity with the negative regulators of anthocyanin/proanthocyanin accumulation such as peach PpMYB18 and poplar PtMYB165 (Fig. 2A). It contains characteristic R2 and R3 domains in its N-terminal region, and the EAR repressive motif in its C-terminal region (Fig. 2B). Subcellular localization assays revealed that PyMYB107 is a nuclei-localized protein (Fig. 2C).

### PyMYB107 represses anthocyanin biosynthesis in pear and Micro-Tom tomato

**Fig. 3:**
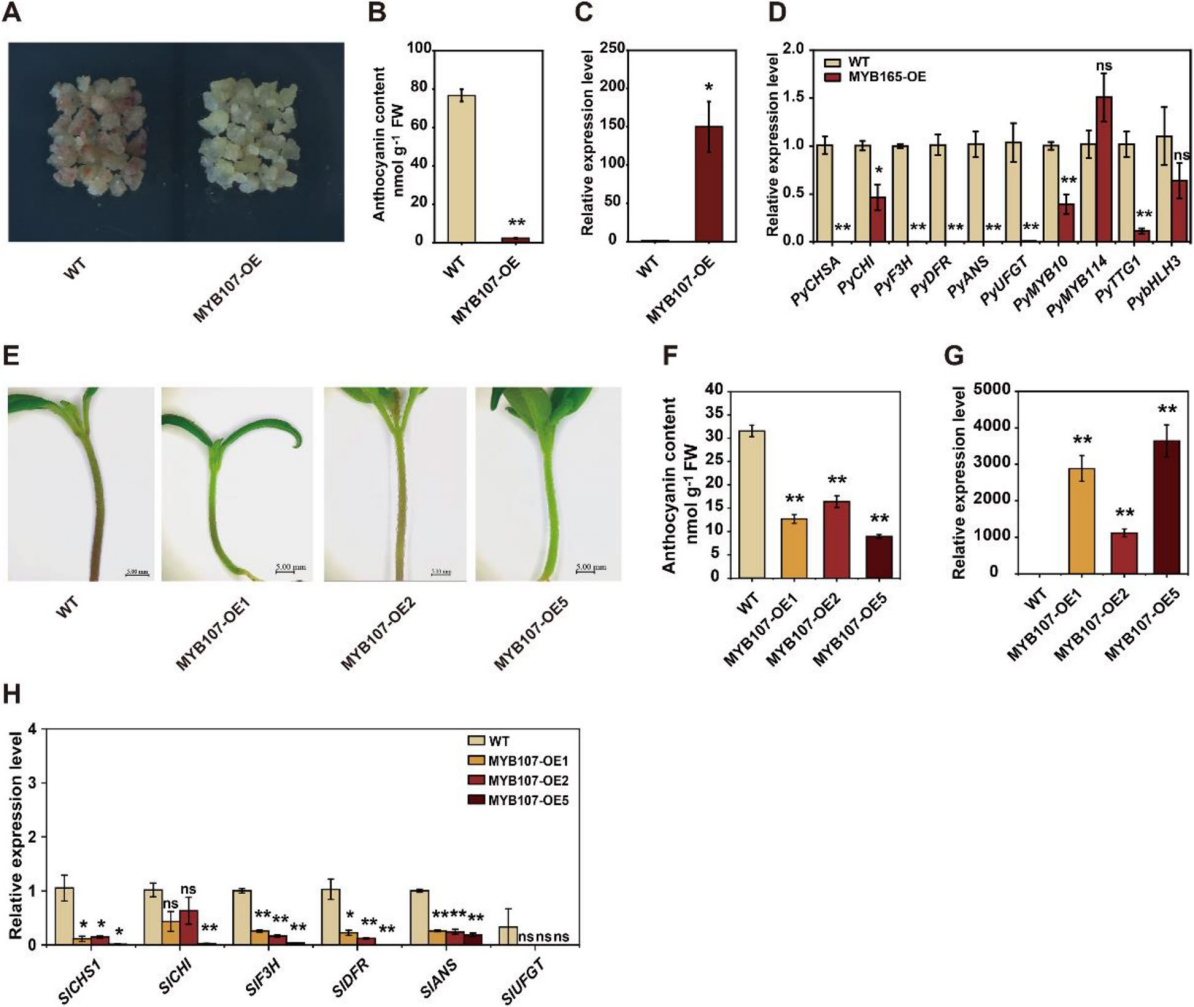
Genetic transformation of *PyMYB107* in pear calli and tomato. **A** Genetic transformation of *PyMYB107* in ‘Clapp’s Favorite’ pear calli. The wild-type calli was used as the control. WT, wild-type. Pear calli was treated in an illumination incubator under continuous light for four days (white/blue light = 15,000 lx/1600 lx). WT, wild-type. **B** Anthocyanin concentration in WT and transgenic pear calli. FW, fresh weight. Error bars represent mean ± SE of three biological replicates. **C** and **D** Expression level of *PyMYB107* and the key anthocyanin-related genes in WT and transgenic pear calli analyzed based-on RT-qPCR. Error bars indicate mean ± SE of three biological replicates. **E** Stable transformation of *PyMYB107* in Micro-Tom tomato. Three transgenic lines showed a reduction in red pigmentation in the seedlings. **F** Anthocyanin concentration in WT and transgenic tomato plants. Error bars represent mean ± SE of three biological replicates. **G **and **H** Expression level of *PyMYB107* and the tomato anthocyanin biosynthetic genes in WT and transgenic tomato plants. Error bars represent mean ± SE of three biological replicates. Asterisks show significant difference based on two-tailed Student’s *t*-test (*P < 0.05, **P < 0.01)

Stable transformation of *PyMYB107* in pear calli led to reduced anthocyanin accumulation after light treatment compared to wild type (WT) calli (Fig. 3A and B). We confirmed the elevated expression level of *PyMYB107*, and expression level of the anthocyanin-related genes including *PyCHSA*, *PyCHI*, *PyF3H*, *PyDFR*, *PyANS*, *PyUFGT* and TF *PyMYB10* were significantly reduced in transgenic pear calli overexpressing PyMYB107 as compared to WT (Fig. 3C and D). We transformed the Micro-Tom tomato with the overexpression construct 35S::PyMYB107, and observed that PyMYB107 transgenic tomato seedlings exhibited a significantly reduced in anthocyanin content as compared to WT (Fig. 3E and F). We observed the expression of *PyMYB107* increased, while the transcripts of some homologous anthocyanin biosynthetic genes in tomato plants significantly decreased (Fig. 3G and H).

**Fig. 4:**
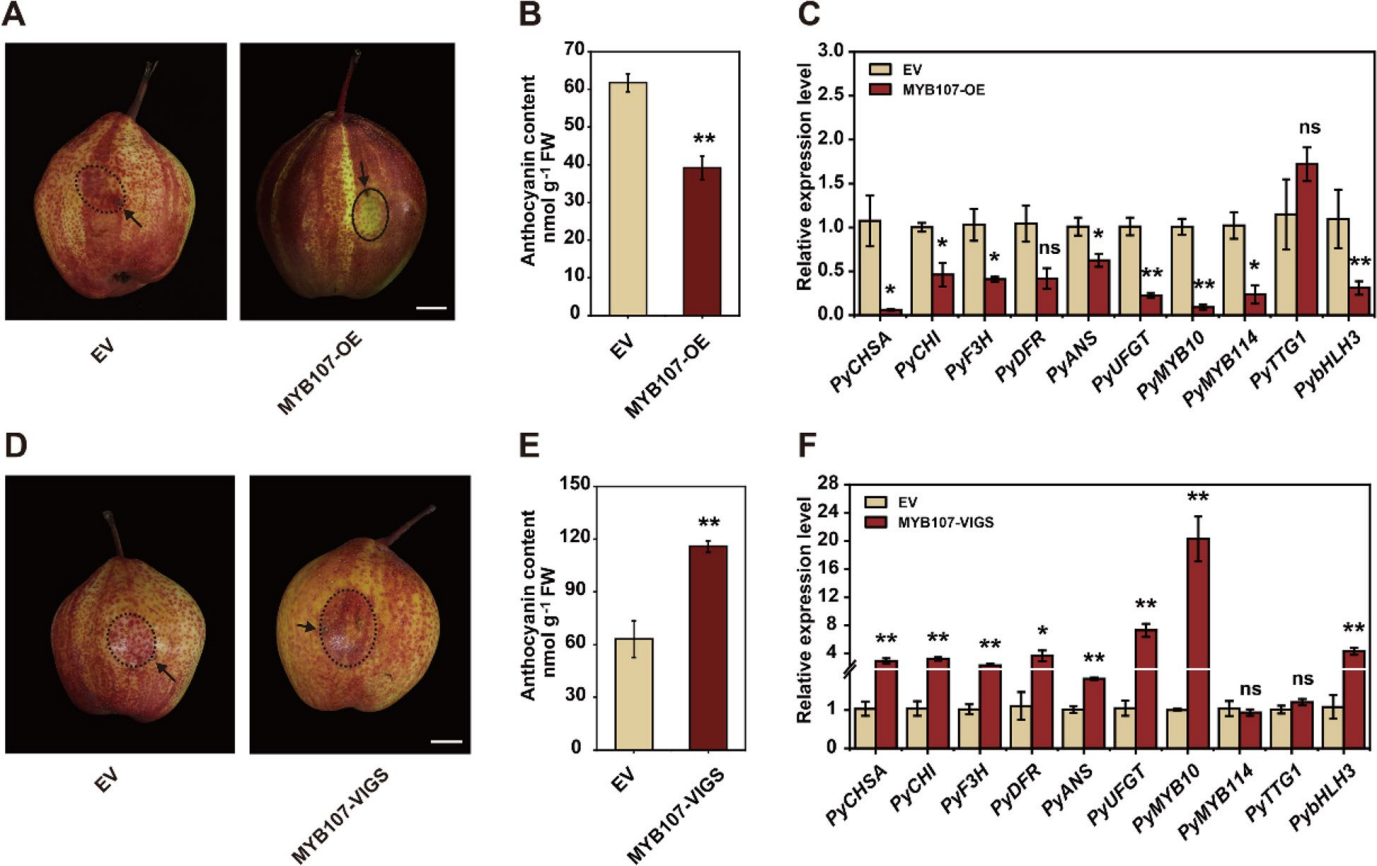
Transient expression of *PyMYB107* in pear fruits. **A** Transient overexpression of *PyMYB107* in ‘Red Zaosu’ pear fruits. Transgenic pear fruits were imaged at 7 d post infiltration. The infiltrated region was circled with the black dotted line. EV, the empty vector pSAK277, used as the control. OE, overexpression. **B** Anthocyanin concentration in EV and PyMYB107-OE pear fruits based on spectrophotometer. FW, fresh weight. **C** Expression level of the anthocyanin-related genes in EV and PyMYB107-OE pear fruits as determined by RT-qPCR. **D** Transient silencing of *PyMYB107* in ‘Red Zaosu’ pear fruits. EV, the empty vectors TRV1 and TRV2, used as the control. VIGS, virus-induced gene silencing. **E** Anthocyanin concentration in control and PyMYB107-VIGS pear fruits. **F** Expression pattern of the anthocyanin-related genes in the control and PyMYB107-VIGS pear fruits. ns, not significant. Asterisks indicate significant difference based on two-tailed Student’s *t*-test (*P < 0.05, **P < 0.01)

To further investigate the effect of PyMYB107 on anthocyanin accumulate, the overexpression construct 35S::PyMYB107 and virus-induced gene silencing (VIGS) construct PyMYB107-VIGS were agro-infiltrated into ‘Red Zaosu’ pear fruits. The expression of *PyMYB107* was confirmed at 7 d after inoculation (Fig. S1, A and B). Overexpression of *PyMYB107* significantly decreased anthocyanin concentration compared with the control, accompanied by a reduction in expression levels of the key anthocyanin-related genes, including *PyANS*, *PyUFGT*, *PyMYB10* and *PyMYB114* (Fig. 4A to C). Conversely, silencing of *PyMYB107* led to a dramatic increase in anthocyanin concentration and expression level of several key anthocyanin-related genes (Fig. 4D to F). These findings strongly indicate that PyMYB107 exerts a repressive role in anthocyanin biosynthesis.

### PyMYB107 inhibits anthocyanin accumulation via transcriptional repression of the activity of *PyANS *and *PyUFGT*

**Fig. 5:**
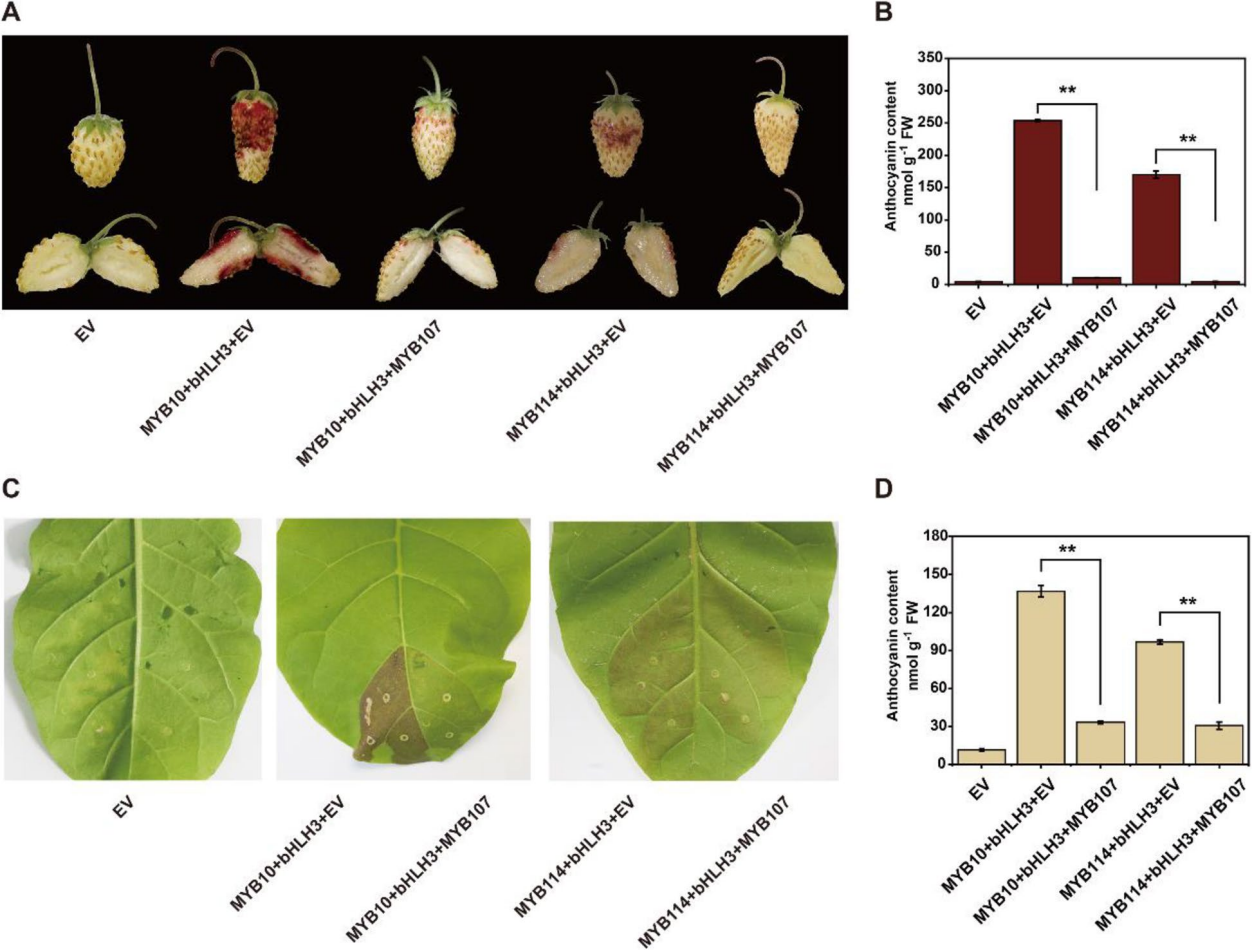
Ectopic overexpression of *PyMYB107* reduces anthocyanin accumulation in strawberry fruits and tobacco leaves. **A** Transient *PyMYB107* overexpression in strawberry fruits. **B** Anthocyanin concentration in the infiltrated strawberry fruits. **C** Transient color assay of the *PyMYB107* activity in tobacco leaves. The images were taken at 7 d post inoculation. **D** Anthocyanin concentration in the infiltrated tobacco leaves. EV, empty vector pSAK277, used as the negative control. *PyMYB10*/*PybHLH3* and *PyMYB114*/*PybHLH3* served as the positive control, respectively. Error bars denote mean ± SE of three biological replicates. Asterisks represent significant difference based on two-tailed Student’s *t*-test (**P < 0.01)

We previously showed that co-infiltration of *PyMYB10* or *PyMYB114* with *PybHLH3* induced anthocyanin biosynthesis in tobacco leaves and strawberry fruits (Yao et al. 2017). Here, we observed a dramatic decrease in anthocyanin concentration, both in tobacco leaves and strawberry fruits when *PyMYB107* was co-infiltrated with *PyMYB10* or *PyMYB114* with *PybHLH3* (Fig. 5A to D), indicating that PyMYB107 suppressed anthocyanin biosynthesis activation by PyMYB10/bHLH3 or PyMYB114/bHLH3.

**Fig. 6:**
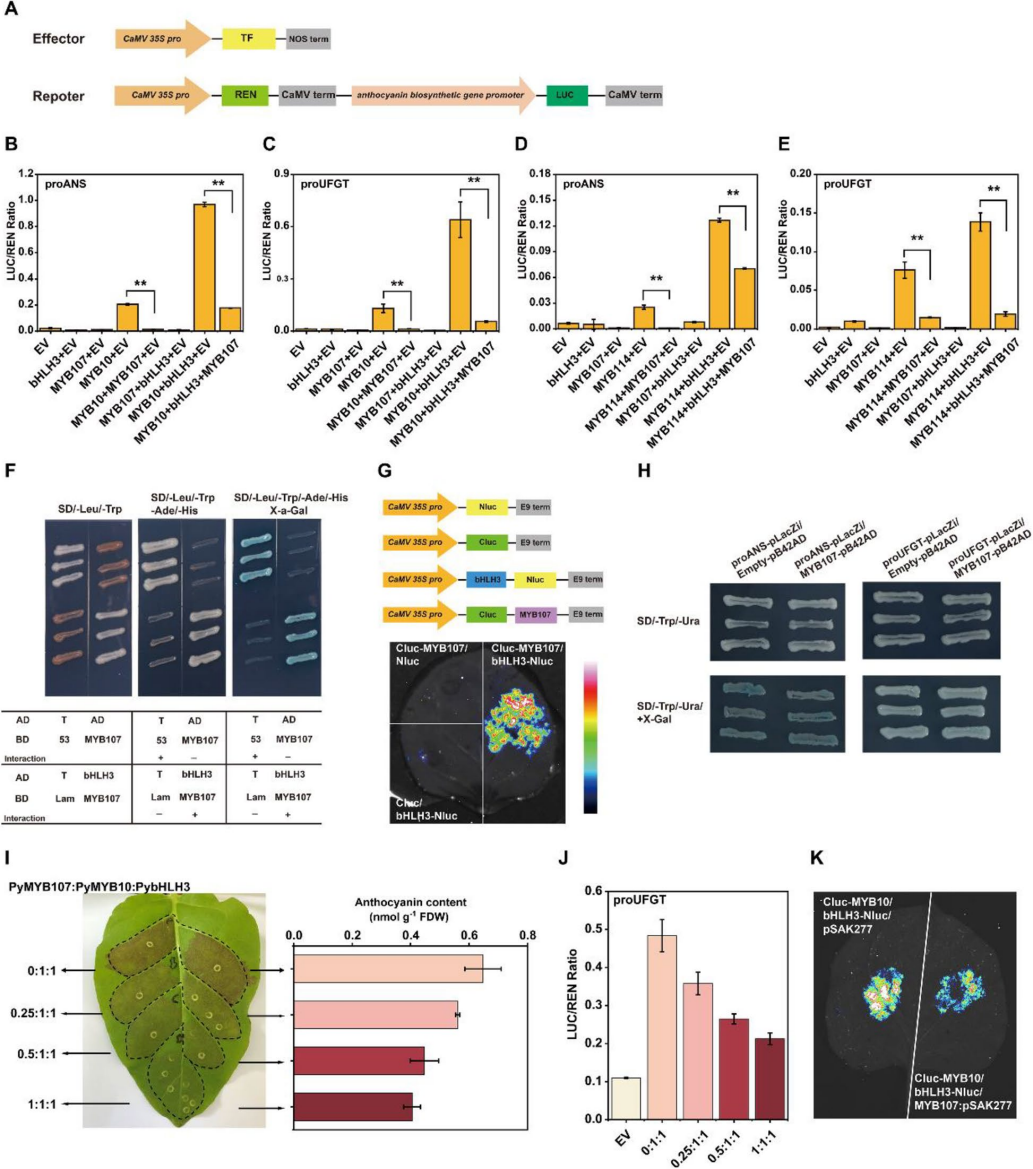
PyMYB107 interacts with PybHLH3 to suppress the activity of *PyANS* and *PyUFGT.***A** Schematic diagram showing the effector and reporter constructs for the dual-luciferase assay. **B-E** Dual-luciferase assay to detect the activity of firefly luciferase (LUC) and renilla luciferase (REN), testing effect of PyMYB107 on the transcription of *PyANS* and *PyUFGT* genes, respectively. Error bars denote mean ± SE of three biological replicates. Asterisks indicate significantly different values as determined by two-tailed Student’s *t*-test (*P < 0.05, **P < 0.01). **F** Yeast two-hybrid assay showing the interaction between PyMYB107 and PybHLH3. The yeast cells grew on the SD/-Leu/-Trp, SD/-Leu/-Trp/-/Ade/-His and SD/-Leu/-Trp/-/Ade/-His/ + X-a-Gal medium, respectively. **G** Firefly luciferase complementation assay showing the interaction between PyMYB107 and PybHLH3 in *N*. *benthamiana* leaves. Color bar ranges from blue to purple suggests that the luciferase activity strength is from weak to strong. **H** Yeast one-hybrid assay to detect the interaction between PyMYB107 and the *PyANS* and *PyUFGT* promoters. The yeast cells grew on the SD/-Trp/-Ura and SD/-Trp/-Ura/ + X-Gal medium, respectively. **I** Transient color assays in tobacco leaves by co-expression of *PyMYB107* and *PyMYB10* at different ratios. **J** Activation effect on the *PyUFGT* promoter by co-expression of *PyMYB10* and *PyMYB107* at different ratios. **K** Firefly luciferase complementation imaging assays to show the interaction affinities of PyMYB10 and PybHLH3 when co-expressed with PyMYB107

To unravel the regulatory mechanism of anthocyanin biosynthesis by PyMYB107, the promoters of two key anthocyanin biosynthetic genes, *PyANS* and *PyUFGT,* were cloned into a dual luciferase reporter system (Fig. 6A) to evaluate the influence of PyMYB107 on their transcriptional activity. We found that overexpression of PyMYB107 alone showed no influence on the expression of *PyANS* and *PyUFGT*. Infiltration of *PyMYB10* alone or co-infiltration of *PyMYB10* and *PybHLH3* was able to enhance the transcriptional activity of *PyANS* and *PyUFGT*, while the activation was dramatically suppressed when *PyMYB107* was co-infiltrated with *PyMYB10* and *PyMYB10*/*bHLH3*, respectively (Fig. 6B and C). Similar results were observed when *PyMYB107* was co-transformed with *PyMYB114* and *PyMYB114*/*bHLH3*, respectively (Fig. 6D and E). These results suggest that PyMYB107 suppresses the transcriptional activation of anthocyanin biosynthetic genes through PyMYB10/bHLH3 and PyMYB114/bHLH3.

### PyMYB107 interacts with PybHLH3

R2R3-MYB repressors typically compete with MYB activators for binding to bHLH proteins (Yoshida et al. 2015; Zhou et al. 2019). To elucidate the interaction between PyMYB107 and PybHLH3, we conducted yeast two-hybrid assays. The PyMYB107 protein was fused with GAL4BD as a ‘bait’, while the PybHLH3 protein was fused with GAL4AD as a ‘prey’. Yeast cells co-transformed with PyMYB107-BD and empty AD vector could grow normally on the medium SD/-Trp/-Leu but failed to grow on the medium SD/-Trp/-Leu/-Ade/-His (Fig. 6F). In contrast, yeast cells co-transformed with both PyMYB107-BD and PyBHLH3-AD were capable of normal growth both on SD/-Trp/-Leu and SD/-Trp/-Leu/-Ade/-His medium, and turned blue on SD/-Trp/-Leu/-Ade/-His medium containing X-a-Gal, which indicated an in vitro interaction between PyMYB107 and PybHLH3. Furthermore, a firefly luciferase complementation (FLC) assay confirmed the interaction in vivo between PyMYB107 and PybHLH3 (Fig. 6G).

We further investigated the interaction between PyMYB107 and the promoters of *PyANS* and *PyUFGT* through yeast one-hybrid (Y1H) assays. Yeast cells co-transformed empty pB42AD with *PyANS* and *PyUFGT* promoter served as the control, respectively. As a result, we observed that yeast cells harboring* PyMYB107* and the *PyANS* promoter could be stained pale blue on the SD/-Trp/-Ura medium with X-Gal, while these transformants showed no difference in color compared with the control, indicating that PyMYB107 was unable to directly bind to the promoter of *PyANS*. Additionally, yeast cells carrying* PyMYB107* and the *PyUFGT* promoter were not stained blue on the SD/-Trp/-Ura medium (Fig. 6H), suggesting that PyMYB107 was unable to directly bind to the promoter of *PyUFGT*.

### PyMYB107 inhibits anthocyanin accumulation in a dose-dependent manner

Transient overexpression assays in strawberry fruits and tobacco leaves showed that addition of PyMYB107 attenuated, but did not completely inhibit anthocyanin accumulation induced by the PyMYB10/MYB114-bHLH3 complex (Fig. 5). Hence, the dosage-dependent repression of PyMYB107 was examined using transient overexpression assays. The dosage ratio of *PyMYB107*:*PyMYB10* was set as 0:1, 0.25:1, 0.5:1 and 1:1, respectively, where the ratio of *PybHLH3*:*PyMYB10* remained 1:1. Transient overexpression assays showed that red pigments of tobacco leaves appeared a gradual decline when the ratio of *PyMYB107*:*PyMYB10* ranged from 0:1 to 1:1 (Fig. 6I). Moreover, dual-luciferase assays were conducted to verify the dosage-dependent repression of PyMYB107. We observed that transcriptional activation of the *PyUFGT* promoter reduced by co-infiltration of *PyMYB107* and *PyMYB10* together with PybHLH3, and the value of LUC/REN ratio declined from the average of 0.48 to 0.26 when the ratio of *PyMYB107*:*PyMYB10* ranged from 0:1 to 1:1 (Fig. 6J). These suggest that anthocyanin accumulation of tobacco leaves was controlled via a dose-dependent manner through the ‘activator—repressor’ loop, and anthocyanin concentration was negatively correlated to the amount of PyMYB107. Furthermore, the LCI assay was conducted to confirmed that addition of PyMYB107 diminished interaction affinity of the PyMYB10-PybHLH3 complex compared with addition of the empty vector control (Fig. 6K).

### Effects of EAR motif and R3 domain on the repressive activity of PyMYB107

**Fig. 7:**
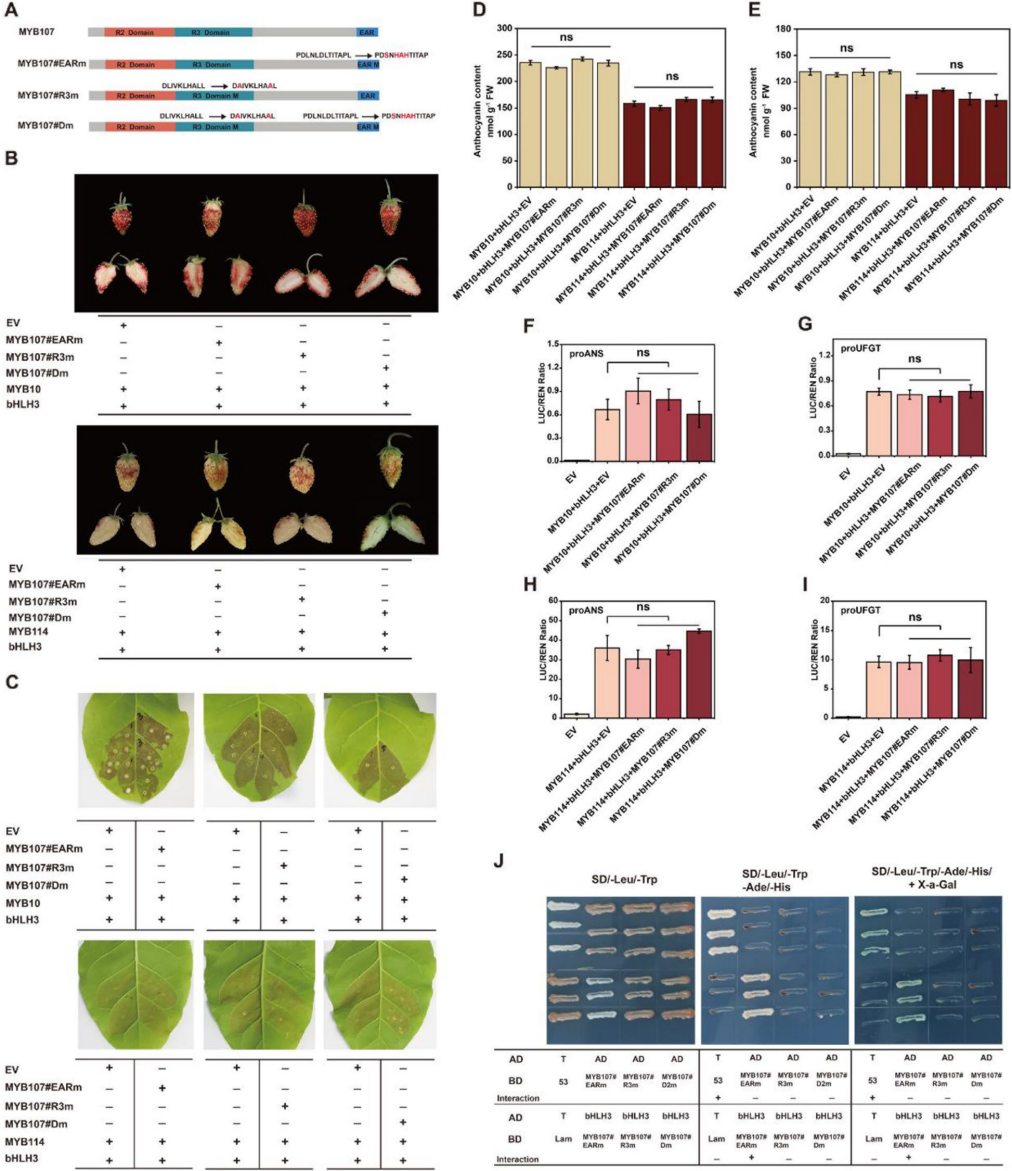
Association of the EAR motif and R3 domain with the repressive activity of PyMYB107. **A** Schematic diagram of PyMYB107 protein and its variants. **B** Phenotype of transiently transformed strawberry fruits with mutated versions of *PyMYB107,* co-infiltrated with *PyMYB10* or *PyMYB114* and *PybHLH3*. **C** Transient color assay in tobacco leaves with mutated versions of *PyMYB107* co-infiltrated with *PyMYB10* or *PyMYB114* and *PybHLH3*. **D** and **E**) Anthocyanin concentration in the infiltrated strawberry fruits **D**) and tobacco leaves **E**). Error bars donate mean ± SE of three biological replicates. ns, not significant. **F** and **I** Dual-luciferase assay showing the activity of *PyANS* and *PyUFGT* promoters with co-transformation of *PyMYB107* and *PyMYB10/PybHLH3* or *PyMYB114/PybHLH3*. Error bars indicate mean ± SE of three biological replicates. ns, not significant. **J** Yeast two-hybrid assay showing the interaction between PyMYB107#EARm, PyMYB107#R3m and PyMYB107#Dm with PybHLH3, respectively

Based on the above findings, we propose that PyMYB107 physically interacts with bHLH3 to interfere with the stability of MBW complex, thereby suppressing anthocyanin accumulation. To elucidate the effects of the EAR motif and R3 domain on the repressive activity of PyMYB107, we generated three variants, namely PyMYB107#EARm, PyMYB107#R3m and PyMYB107#Dm, respectively (Fig. 7A). The residues in the EAR motif and R3 domain were substituted based on prior report (Zhou et al. 2019). Specifically, PyMYB107#EARm variant contained four amino acid substitutions of Leu, Leu, Asp, and Leu with Ser, His, Ala and His at residue positions 192, 194, 195 and 196 within the EAR motif, respectively. PyMYB107#R3m variant showed the single amino acid substitution of Leu with Ala at two residue positions 76 and 83 within the R3 domain, respectively. PyMYB107#Dm protein is a variant harboring the same mutations in both the R3 domain and the EAR motif. Transient color assays revealed that co-expression of *PyMYB10* and *PyMYB114*, respectively, with *PybHLH3* led to intense red pigment in strawberry fruits, while there was no significant change in fruit color phenotype after co-expression of these three variants of *PyMYB107* with *PyMYB10* and *PyMYB114*, respectively, with *PybHLH3*(Fig. 7B). Similar results were observed when these three variants were co-infiltrated with *PyMYB10* and *PyMYB114*, respectively, with *PybHLH3* in tobacco leaves (Fig. 7C). The anthocyanin in concentration strawberry fruits and tobacco leaves were observed to be consistent with their color phenotypes (Fig. 7D and E). These findings suggest that both the EAR motif and R3 domain play crucial roles in conferring the repressive activity of PyMYB107.

Next, a dual-luciferase assay showed that three variants of *PyMYB107* did not reduce the transcriptional activity on the *PyANS* and *PyUFGT* induced by PyMYB10/bHLH3 complex when they were co-infiltrated with *PyMYB10*/*bHLH3*, respectively (Fig. 7F and G). Similar results were observed when three variants of *PyMYB107* were co-infiltrated* PyMYB114*/*bHLH3*, respectively. We further examined the interaction between the three variants of PyMYB107 with PybHLH3. Yeast two-hybrid assays demonstrated that PyMYB107#EARm was able to interact with PybHLH3. In contrast, PyMYB107#R3m and PyMYB107#Dm, which lack a functional bHLH binding site, lost their ability to interact with PybHLH3 (Fig. 7J). In addition, the three variants of PyMYB107 were located in the nuclei, suggesting that mutations of EAR motif and R3 domain did not alter its subcellular location (Fig. S2).

### *PyMYB107* is transcriptionally activated by PyMYB10 and PyMYB114

*PyMYB107* exhibited a similar expression pattern to *PyMYB10* and *PyMYB114* between ‘Zaosu’ and ‘Red Zaosu’ pear (Fig. 1D), suggesting their potential influence on the transcription of *PyMYB107*. Furthermore, a dual-luciferase assay was conducted, and the result showed that both PyMYB10 and PyMYB114 could activate the expression of *PyMYB107* (Fig. S3), indicating PyMYB107 acts as a downstream of PyMYB10 and PyMYB114 activators.

## Discussion

### PyMYB107 is a negative regulator of anthocyanin biosynthesis

R2R3-MYB TFs are involved in a variety of biological processes including phenylpropanoid metabolism, biotic and abiotic stresses, differentiation and hormonal response (Ambawat et al. 2013). R2R3-MYB members in SG4 and SG6 are capable of participating in the transcriptional repression and activation of anthocyanin biosynthesis, respectively (Li et al. 2016; Song et al. 2020; Ni et al. 2021). The reported activators PyMYB10, PyMYB114, PuMYB110 and PbMYB9 are classified as members of SG6 (Feng et al. 2010; Zhai et al. 2016; Yao et al. 2017, 2023; Liu et al. 2021), while the repressors, PyMYB120 and PyMYB140, belong to SG4 (Song et al. 2020; Ni et al. 2021).

In this study, we identified PyMYB107, a member of SG4 (Li et al. 2016), as a R2R3-MYB repressor. PyMYB107 showed significant differential expression in ‘Zaosu’ and ‘Red Zaosu’ pears (Fig. 1D). Overexpression of *PyMYB107* reduced anthocyanin biosynthesis in pear calli and pear fruits, whereas transient silencing of PyMYB107 led to increase in anthocyanin concentration in pear fruits (Fig. 3, 4). Moreover, ectopic overexpression of *PyMYB107* reduced anthocyanin accumulation in tomato seedlings, strawberry fruits and tobacco leaves (Figs. 3, 5). PyMYB107 downregulated the expressions of the anthocyanin biosynthetic genes in pear, including *PyCHSA*, *PyCHI*, *PyF3H*, *PyDFR*, *PyANS* and *PyUFGT*, consistent with previous studies that MYB genes regulate the anthocyanin biosynthesis pathway (Allan et al. 2008; Allan and Espley 2018). Previously, we showed that co-overexpression of PyMYB10 or PyMYB114 with PybHLH3 activated anthocyanin accumulation in tobacco leaves and strawberry fruits (Yao et al. 2017), while in the present study, we found that their activation on the *PyANS* and *PyUFGT* was suppressed by PyMYB107 (Fig. 6B to E). PyMYB107 is a homolog of peach PpMYB18 (Fig. 2A and B). Overexpression of *PpMYB18* suppressed the anthocyanin and proanthocyanin biosynthesis in tobacco leaves and *Arabidopsis* seeds to avoid their overaccumulation, and our findings from transient color assays in tobacco leaves were consistent with this reported (Zhou et al. 2019).

### PyMYB107 interacts with activators PyMYB10 and PyMYB114 to form an ‘activator-repressor’ loop

Although PyMYB107 functions as a repressor, it did not completely suppress anthocyanin accumulation. Increase amount of PyMYB107 enhanced its competitive binding ability with PyMYB10 to PybHLH3, and attenuated the transcriptional activation of PyMYB10 on the *PyUFGT*, and ultimately resulting in a reduction in anthocyanin accumulation (Fig. 6). By using transient assays, we found that PyMYB107 was able to reduce anthocyanin concentration that was activated by PyMYB10/PybHLH3 or PyMYB114/PybHLH3 by approximately 50% or more (Fig. 5). In peach, *PpMYB18* expressed differentially between red- and white fleshed fruit during the ripening stage when anthocyanin rapidly accumulated in flesh. Its transcription was positively correlated with *PpMYB10.1* and could be activated by PpMYB10.1 (Zhou et al. 2019). In citrus, *CsMYB3* exhibits significantly higher transcription level in red young fruits and leaves, and its expression level was consistent with the activator *CsRuby* (Huang et al. 2019). Similar results were also observed for FaMYB1 in strawberry (Aharoni et al. 2001). Consistent with these reports, *PyMYB107* showed a higher expression level in red-skinned pear fruits, which correlative with the expression profiles of anthocyanin biosynthetic genes and PyMYB10 and PyMYB114 TFs, and could be activated by PyMYB10 and PyMYB114 (Fig. S3).

R2R3-MYB repressors have been reported to hinder flavonoid biosynthesis through two ways: competitive with MBW complex for binding to bHLH proteins to interfere with MBW complex stability; direct binding to *cis*-acting elements on promoters of the flavonoid biosynthetic genes to repress their transcription (Ambawat et al. 2013; Ma and Constabel 2019; LaFountain and Yuan 2021). For instance, PpMYB18 inhibited anthocyanin accumulation through interaction with PpbHLH3 in vivo and in vitro (Zhou et al. 2019). CsMYB3 was capable of interacting with CsbHLH3, and also showed direct binding to the promoters of anthocyanin biosynthetic genes *CsF3’H* and *CsDFR* (Huang et al. 2019). In the present study, PyMYB107 showed interaction with PybHLH3 both in vivo and in vitro (Fig. 6F and G), whereas it could not directly bind to the *PyANS* and *PyUFGT* promoters (Fig. 6H), suggesting that their functions produce similar effects, but they differ in the regulatory mechanisms.

**Fig. 8:**
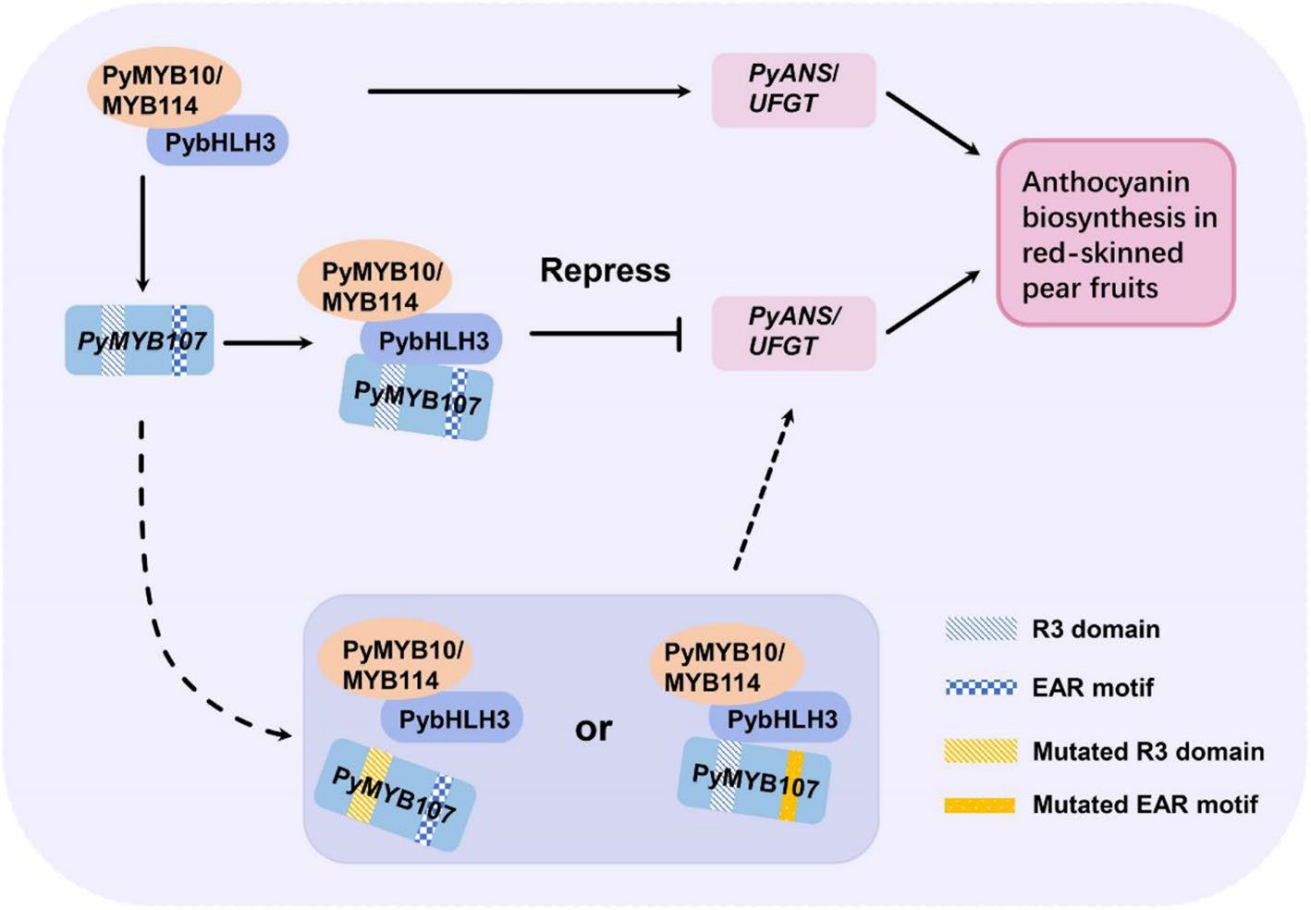
A proposed model of PyMYB107 in the regulation of anthocyanin biosynthesis in pear fruits. In the model, the complex PyMYB10/MYB114-PybHLH3 activates the key anthocyanin biosynthetic genes *PyANS*/*UFGT* and *PyMYB107* gene, while PyMYB107 in turn competes with PyMYB10 and PyMYB114 to bind the PybHLH3 protein, interfering with the stability of the MBW complex, thereby repressing excessive accumulation of anthocyanin. However, when R3 domain or EAR motif was mutated, the repressive activity of PyMYB107 was inactivated, and anthocyanin can continue to accumulate

Together, we proposed that PyMYB107 competes with PyMYB10 and PyMYB114 to interact with bHLH3 through its R3 domain, while *PyMYB107* could be activated by PyMYB10 and PyMYB114 to form a regulatory as ‘activator-repressor’, thereby balancing anthocyanin accumulation. In the loop, PyMYB114, could, but PyMYB10 did not, directly bind the *PyMYB107* promoter (Fig. 8), suggesting that distinct mechanisms by which both PyMYB114 and PyMYB10 regulate the expression of *PyMYB107*. However, further validation is needed to fully elucidate these mechanisms.

### Influence of R3 domain and EAR motif on the repressive function of PyMYB107

The EAR motif, characterized by the consensus sequence LxLxL or DLNxxP, is a widely distributed plant inhibition motif. Proteins containing this motif are often involved in plant hormone signaling or stress response pathways that aid in recruiting chromatin remodeling factors for gene expression regulation (Kagale and Rozwadowski 2011). In *Arabidopsis*, deletion or mutation of the EAR motif from AtMYBL2 and PtMYB182 did not affect its repressive activity on anthocyanin accumulation (Matsui et al. 2008; Yoshida et al. 2015). In contrast, two types of mutations in the EAR motif of PpMYB18 resulted in partial or complete loss of its repressive activity (Zhou et al. 2019). In pear, the function of the R3 domain and EAR motif of the MYB repressors on the repressive activity have not been well elaborated, despite PbMYB120 and PpMYB140 being known to inhibit anthocyanin accumulation (Song et al. 2020; Ni et al. 2021). Recently, it has been reported that PyERF4.1/PyERF4.2 interacted with PyERF3 to affect the stability of the PyERF3-PyMYB114-PybHLH3 complex to suppress the expression of the anthocyanin biosynthesis gene *PyANS*. Deletion of the EAR motif eliminated the repressive activity of PyERF4.1/PyERF4.2 on anthocyanin biosynthesis, while a mutation of the PyERF4.2-EAR motif enhanced its repressive activity (Sun et al. 2023). In the present study, we clarified the molecular regulatory mechanism of PyMYB107 involved in the regulation of anthocyanin accumulation by employing a series of experiments, and clarified their crucial role of the EAR motif and R3 domain in its repressive activity (Figs. 3, 4, 5, 6 and 7). Consistent with findings for PpMYB18 (Zhou et al. 2019), we showed that four single amino acid mutations within the EAR motif of PyMYB107 led to deactivation of its repressive activity (Fig. 7).

The R3 domain is essential for interaction with bHLH protein (Ma and Constabel 2019), which has been confirmed through yeast two-hybrid assays (Xiang et al. 2019; Zhou et al. 2019). We validated that PyMYB107 was able to interact with PybHLH3; however, PyMYB107 lost this ability when it lacked a functional bHLH binding motif (Fig. 7), which provided additional evidence and agreed with the previous reports (Xiang et al. 2019; Zhou et al. 2019). PyMYB107#R3m or PyMYB107#Dm, when co-overexpressed with PyMYB10/MYB114 and PybHLH3, showed no significant change in anthocyanin concentration in tobacco leaves and strawberry fruits, and did not remarkably affect the transcriptional activity of *PyANS* and *PyUFGT*. These further demonstrate that the intact bHLH binding motif within R3 domain is required for the repressive activity of PyMYB107 of anthocyanin accumulation.

## Materials and methods

### Plant materials and growth conditions

Pear fruit samples of ‘Zaosu’ and ‘Red Zaosu’ were collected from Liyuan Bay in Suqian City, Jiangsu Province. Pear fruits at 30 days after flower bloom (DAFB) were used for color phenotype analysis. ‘Yellow Wonder’ 5AF7 (YW5AF7) (*F. vesca*) strawberry, ‘Micro-Tom’ tomato, ‘Samsun’ tobacco (*N. tabacum*) and *N. benthamiana* grew in a greenhouse at 22 ± 2℃ with a photoperiod of 16/8 h light/dark. The ‘Clapp Favorite’ pear calli grew on tissue culture medium under the dark at 22 ± 2℃.

### Extraction of the genomic DNA and total RNA, and RT-qPCR

Plant genomic DNA (gDNA) was extracted using the Plant DNA Extraction Kit (Vazyme, China) and plant total RNA was extracted using the SpectroTM Plant Total RNA Kit (Sigma, USA), the extraction procedures were according to the manufacturer instructions, respectively. A total of 1000 ng of RNA was used for the first-strand cDNA synthesis using the HiScript II Reverse Transcriptase Kit (Vazyme, China) containing gDNA remover. Real-time quantitative polymerase chain reaction (RT-qPCR) was performed using 2 × SYBR® Master (Roche, USA). The PCR procedure was performed on a LightCycler 480 real-time PCR system: one cycle at 95 °C for 30 s, followed by forty cycles at 95 °C for 3 s; at 60 °C for 10 s; at 72 °C for 30 s. Transcription levels were analyzed using the ΔΔC_t_ method as described previously (Livak and Schmittgen 2001).

### Extraction and determination of anthocyanins

The extraction method for anthocyanins was followed the previous study (Yao et al. 2017). In brief, the freshly weighed sample was ground into powder and transferred to a 10 mL centrifuge tube containing 0.1% HCl methanol and then soaked for 24 h at 4 °C in darkness. The supernatant was determined at 530, 620 and 650 nm using UV-1800 spectrophotometer with MAPADA software. Total anthocyanin content (TA) was calculated as TA = ODλ × V × 10^6^/(ελ × T), where Dλ = (OD530-OD620)-0.1(OD650-OD620), TA represents the total anthocyanin content in nanomoles per gram (nmol g^−1^), V is the final volume in milliliters (mL), T is the sample mass in grams (g), ελ = 4.62 × 10^4^ is the molar extinction coefficient at wavelength λ = 530 nm. Three biological replicates were performed for each treatment.

### Gene isolation and vector construction

Gene isolation and vector construction were carried out according to the instructions provided by Phanta SE Super-Fidelity DNA Polymerase Kit (Vazyme) and ClonExpress Ultra One Step Cloning Kit (Vazyme). The cDNA and gDNA from ‘Red Zaosu’ pear fruit skin was used as templates for cloning based on the specific primers (Table S1).

### Subcellular localization

The full-length coding region of *PyMYB107* and its variants were amplified and inserted into pCAMBIA1301 vector containing the green fluorescent protein (GFP) to generate 35S::PyMYB107-GFP, 35S::PyMYB107#EARm-GFP, 35S::PyMYB107#R3m-GFP and 35S::PyMYB107#Dm-GFP constructs, respectively. Then, these constructs were transformed into *A. tumefacien* strain GV3101. *Agrobacterium* harboring the construct was suspended in the infiltration solution containing 20 mM MgCl_2_, 20 mM MES (pH 5.6) and 150 μM acetylsyringone, OD600 = 0.8. Leaves of 3-week-old *N. benthamiana* were used for infiltration. At 3 days post-infiltration, the leaves of *N. benthamiana* were collected and placed under a laser confocal microscope (Leica, Germany) to check fluorescence signals. ZEN 2012 (CarlZeiss, Germany) software was used to process images.

### Genetic transformation of pear calli

Pear calli is derived from ‘Clapp’s Favorite’ (*P. communis*) cultivar. The 35S::PyMYB107-GFP construct was transformed into *A. tumefacien* strain EHA105 for genetic transformation of pear calli based on the previous study with minor modifications (Bai et al. 2019). The transgenic pear calli was selected on the screening MS medium with 20 mg L^−1^ hygromycin resistant and cultured at 24℃ in darkness and the positive transgenic calli was confirmed using RT-qPCR. *PyActin* was used as the housekeeping gene. For light treatment, transgenic calli was placed in an incubator with continuous light (white light/blue light = 15,000 lx /1600 lx) for 4 d, and then the calli was imaged and harvested for further analysis.

### Stable transformation of tomato

35S::PyMYB107 was transformed into *A. tumefacien* strain EHA105 for Micro-Tom tomato transformation. The transformation used the *Agrobacterium*-mediated method conducted according to previous studies with modifications (Chetty et al. 2013). In brief, the pre-cultural hypocotyl and cotyledon explants of tomato seedings were inoculated in the Agrobacterium suspension for approximately 20 min, and inoculated explants were dried on sterile filter paper and then transferred to co-culture medium for 2 d. After that, explants were placed onto shoot induction medium containing 4.74 g L^−1^ MS, 30 g L^−1^ sucrose, 2 mg L^−1^ ZT, 0.5 mg L^−1^ IAA, 300 mg L^−1^ timentin, 100 mg L^−1^ kanamycin and 0.75% phytagel. The regenerated shoots were then transferred onto the root induction medium containing 4.74 g L^−1^ MS, 30 g L^−1^sucrose, 0.5 mg L^−1^ IAA, 300 mg L^−1^ timentin, 100 mg L^−1^ kanamycin and 0.75% phytagel. The T_0_ transgenic tomato lines were confirmed by RT-qPCR and used for phenotype analysis. *SlActin* gene was used as the internal control.

### Transient expression in pear, strawberry fruits and tobacco leaves

‘Red Zaosu’ pear fruits at approximately 105 DAFB, ‘Yellow Wonder’ 5AF7 (YW5AF7) (*F. vesca*) strawberry fruits and leaves of ‘Samsun’ tobacco (*N. tabacum*) were used for transient expression assays. *Agrobacterium*-mediated transformation was used for transient expression assays according to the previous report (Yao et al. 2017) with minor modifications. The ORFs of TFs were ligated into pSAK277 vector to create 35S::PyMYB10, 35S::PyMYB114 and 35S::PybHLH3 constructs for transient overexpression assays, and the specific coding region of *PyMYB107* was ligated into TRV2 vector to create PyMYB107-VIGS construct for transient silencing assay. These constructs were transformed into *A. tumefacien* strain GV3101. Briefly, the Agrobacterium harboring the constructs was suspended into a 50 mL centrifuge tube using the infiltration buffer (mentioned in Subcellular localization), and incubated for 2 h. Then, the suspension was injected in pear fruits, strawberry fruits and tobacco leaves using 1 mL syringes. Three fruits or one leaf were used as one replicates, and three replicates were performed. Transgenic samples were imaged and collected for further analysis at 7 days after inoculation.

### Dual-luciferase assay

The promoter regions of *PyANS*, *PyUFGT* and *PyMYB107* were inserted into pGreenII 0800-LUC vector to generate proANS, proUFGT and proMYB107 plasmids, respectively, then they were transformed into *A. tumefacien* strain GV3101 with pSoup. These constructs and 35S::PyMYB107, 35S::PyMYB10, 35S::PyMYB114 and 35S::PybHLH3 constructs were used for the dual-luciferase assay based on the previous reports (Lin-Wang et al. 2010; Yao et al. 2017). Leaves of 3 to 4-week-old *N. benthamiana* were used for infiltration. At 4 d post infiltration, leaf samples were collected for detecting the activity of Firefly luciferase (LUC) and Renilla luciferase (REN) using Dual-Luciferase® Reporter Assay System (Promega, USA).

### Yeast two-hybrid

The ORFs of *PyMYB107* and *PybHLH3* were ligated into pGBKT7 and pGADT7 vectors to produce PyMYB107-BK and PybHLH3-AD constructs, respectively, for the yeast two-hybrid. The polyethylene glycol/lithium acetate transformation method, as described in the Matchmaker™GAL4 two-hybrid System (Clontech) user manual with minor adjustments, was employed. Briefly, equal amounts of recombinant plasmids PyMYB107-BK and PybHLH3-AD and 10 μg salmon sperm DNA were co-transformed into yeast strain AH109. After transformation, the yeast strain was sprayed onto the SD/-Trp/-Leu and incubated at 28℃ for 3 d. Then, the sing colony of yeast cells was striped onto the SD/-Trp/-Leu/-Ade/-His and SD/-Trp/-Leu/-Ade/-His/ + X-a-Gal media and incubated at 28℃ for another 3–5 d.

### Luciferase complementary assay

The coding regions of *PyMYB107* and *PybHLH3* were inserted into Cluc and Nluc vectors to generate Cluc: PyMYB107 and PybHLH3: Nluc constructs, respectively. Luciferase complementary assay was conducted following the subcellular localization. The transgenic leaves of *N. benthamiana* were collected at 3 days after infiltration. A solution of luciferin potassium salt (1 mM) was evenly sprayed on the back of the leaves, followed by incubation in darkness for 5 min. Firefly luciferase (LUC) activity was detected and images were captured using a CCD imaging device (Tanon 5200 Multi).

### Yeast one-hybrid

The full-length coding region of *PyMYB107* was ligated into pB42AD to generate PyMYB107-pB42AD. The promoter sequences of *PyANS* (2000 bp) and *PyUFGT* (2000 bp) were ligated into pLacZi vector to produce proANS-pLacZi and proUFGT-pLacZi constructs, respectively. Yeast one-hybrid was performed following the produce of the yeast two-hybrid assay. The yeast strain EGY48 was used for transformation, and the yeast growth media included SD/-Ura/-Trp and SD/-Ura/-Trp/ + X-Gal.

### Statistical analysis

Statistical analysis was performed using Student’s *t*-test (*P < 0.05 and**P < 0.01) and one-way ANOVA (*P* < 0.05). Data analysis was conducted using Microsoft Excel 2019 and GraphPad Prism 6.0.

## Data Availability

The data will be available from the corresponding author upon reasonable request.

## Supplementary Information

Additional file 1: Fig. S1. Expression level of *PyMYB107* in transgenic pear fruits. Additional file 2: Fig. S2 Subcellular localization of PyMYB107#EARm, PyMYB107#R3m and PyMYB107#Dm proteins. Additional file 3: Fig. S3 Dual-luciferase assay to detect the promoter activity of *PyMYB107*. Additional file 4: Table S1 primer sequences used in this study.

## Abbreviations

FLC: Firefly luciferase complementation
GFP: Green fluorescent protein
LUC: Firefly luciferase
ORF: Open reading frame
RT-qPCR: Quantitative real-time polymerase chain reaction
REN: Renilla luciferase
TFs: Transcription factors
VIGS: Virus induced gene silencing
Y1H: Yeast one-hybrid assay
Y2H: Yeast two-hybrid assay
