# Supplementary material for: An ‘activator-repressor’ loop controls the anthocyanin biosynthesis in red-skinned pear
Source: Mol Hortic. 2024 Jul 1;4:26. doi: 10.1186/s43897-024-00102-6 (PMC11215833; doi:10.1186/s43897-024-00102-6)
Supplement: Supplementary file 1 — Additional file 1: Fig. S1. Expression level of PyMYB107 in transgenic pear fruits. [file 43897_2024_102_MOESM1_ESM.pdf]

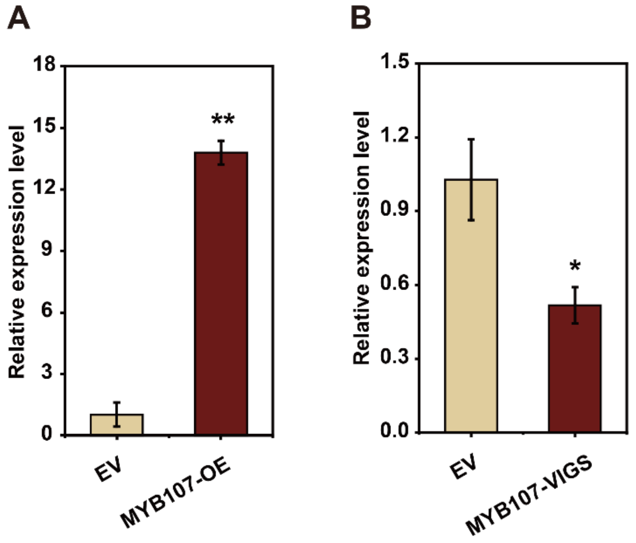

Fig. S1 Expression level of *PyMYB107* in transgenic pear fruits. A and B) Relative expression level of PyMYB107 in the transgenic pear fruits overexpressing *PyMYB107* **(A)** and silencing *PyMYB107* **(B)** as determined by RT-qPCR. EV represents the empty vector pSAK277 in transient overexpression assays, and the vectors TRV1 and TRV2 were used as EV control in transient silencing assays. Error bars show mean ± SE of three biological replicates. Asterisks indicate significant differences based on two-tailed Student's *t*-test (**P*< 0.05, ***P*< 0.01).
