# Supplementary material for: An ‘activator-repressor’ loop controls the anthocyanin biosynthesis in red-skinned pear
Source: Mol Hortic. 2024 Jul 1;4:26. doi: 10.1186/s43897-024-00102-6 (PMC11215833; doi:10.1186/s43897-024-00102-6)
Supplement: Supplementary file 2 — Additional file 2: Fig. S2 Subcellular localization of PyMYB107#EARm, PyMYB107#R3m and PyMYB107#Dm proteins. [file 43897_2024_102_MOESM2_ESM.pdf]

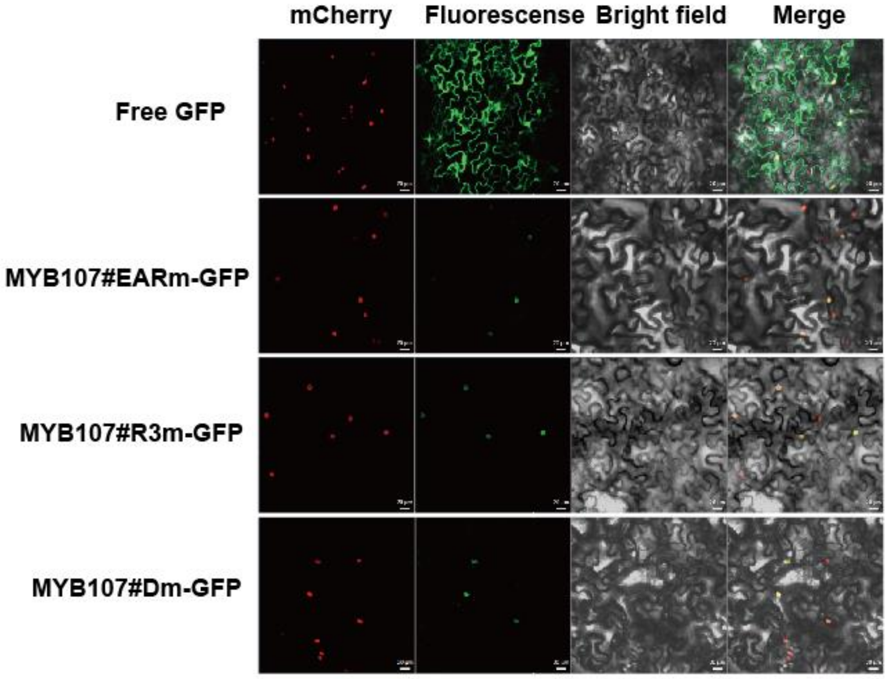

Fig. S2 Subcellular localization of PyMYB107#EARm, PyMYB107#R3m and PyMYB107#Dm proteins. The signals were detected by laser confocal microscopy. Free GFP was used as the control. Bar, 20μm.
