# Supplementary material for: An ‘activator-repressor’ loop controls the anthocyanin biosynthesis in red-skinned pear
Source: Mol Hortic. 2024 Jul 1;4:26. doi: 10.1186/s43897-024-00102-6 (PMC11215833; doi:10.1186/s43897-024-00102-6)
Supplement: Supplementary file 3 — Additional file 3: Fig. S3 Dual-luciferase assay to detect the promoter activity of PyMYB107. [file 43897_2024_102_MOESM3_ESM.pdf]

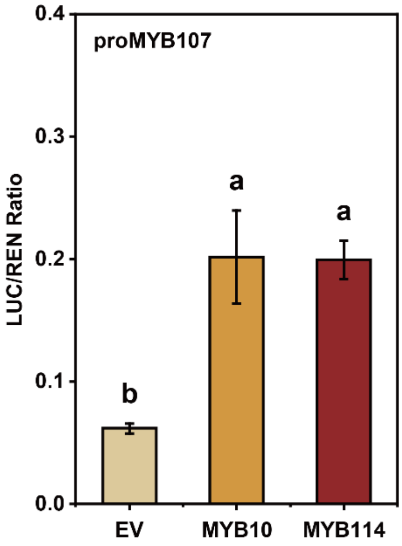

Fig. S3 Dual-luciferase assay to detect the promoter activity of *PyMYB107*. A dual-luciferase assay to detect effect of PyMYB10 and PyMYB114 on the promoter activity of *PyMYB107*. Lowercase letters above the bars show statistical significance based on One-way ANOVA followed by Tukey's multiple comparisons test ($P < 0.05$).
